# Effectiveness of a Mobile App (Meds@HOME) to Improve Medication Safety for Children With Medical Complexity: Protocol for a Randomized Controlled Trial

**DOI:** 10.2196/60621

**Published:** 2024-09-09

**Authors:** Nicole E Werner, Makenzie Morgen, Sophie Kooiman, Anna Jolliff, Gemma Warner, James Feinstein, Michelle Chui, Barbara Katz, Brittany Storhoff, Kristan Sodergren, Ryan Coller

**Affiliations:** 1 Indiana University School of Public Health-Bloomington Bloomington, IN United States; 2 University of Wisconsin-Madison School of Medicine and Public Health Madison, WI United States; 3 University of Colorado School of Medicine Aurora, CO United States; 4 University of Wisconsin-Madison School of Pharmacy Madison, WI United States; 5 Family Voices of Wisconsin Madison, WI United States

**Keywords:** medication safety, children with medical complexity, caregiving, polypharmacy, medication management

## Abstract

**Background:**

This study will pilot-test the mobile app, Medication Safety @HOME—Meds@HOME intervention to improve medication administration accuracy, reduce preventable adverse drug events, and ultimately improve chronic care management for children with medical complexity (CMC). The Meds@HOME app was co-designed with CMC families, secondary caregivers (SCGs), and health professionals to support medication management for primary caregivers (PCGs) and SCGs of CMC. We hypothesize that Meds@HOME will improve caregivers’ medication administration accuracy, reduce preventable adverse drug events, and ultimately improve chronic care management.

**Objective:**

This study aims to evaluate the effectiveness of Meds@HOME on medication administration accuracy for PCGs and SCGs.

**Methods:**

This study will recruit up to 152 PCGs and 304 SCGs of CMC who are prescribed at least 1 scheduled high-risk medication and receive care at the University of Wisconsin American Family Children’s Hospital. PCGs will be randomly assigned, for the 6-month trial, to either the control group (not trialing Meds@HOME) or the intervention group (trialing Meds@HOME) using 1:1 ratio. The Meds@HOME app allows caregivers to create a child profile, store medication and care instructions, and receive reminders for upcoming and overdue care routines and medication refills. Surveys completed both at the start and end of the trial measure demographics, medication delivery knowledge, confidence in the CMC’s caregiving network, and comfort with medical information. Univariate and multivariate generalized estimation equations will be used for primary statistical analysis. The primary outcome is the PCG’s rate of medication administration accuracy measured as correct identification of each of the following for a randomly selected high-risk medication: indication, formulation, dose, frequency, and route at baseline and after 6 months. Secondary outcomes include SCG medication administration accuracy (indication, formulation, dose, frequency, and route), count of University of Wisconsin hospital and emergency department encounters, PCG-reported medication adherence, count of deaths, and PCG medication confidence and understanding.

**Results:**

Recruitment for this study began on November 29, 2023. As of May 15, 2024, we have enrolled 94/152 (62%) PCGs. We expect recruitment to end by August 1, 2024, and the final participant will complete the study by January 28, 2025, at which point we will start analyzing the complete responses. We expect publication of results at the end of 2025.

**Conclusions:**

The Meds@HOME mobile app provides a promising strategy for improving PCG medication safety for CMC who take high-risk medications. In addition, this protocol highlights novel procedures for recruiting SCGs of CMC. In the future, this app could be used more broadly across diverse caregiving networks to navigate complex medication routines and promote medication safety.

**Trial Registration:**

ClinicalTrials.gov NCT05816590; https://clinicaltrials.gov/study/NCT05816590

**International Registered Report Identifier (IRRID):**

DERR1-10.2196/60621

## Introduction

### Background

Medication errors during routine care at home are common among US children, occurring every 8 minutes [1]. Children with medical complexity (CMC), who have multiple chronic conditions, functional limitations, high health services use, and substantial family-identified needs [2], are uniquely vulnerable to medication errors and adverse drug events. Their treatment plans often include high-risk medications having serious potential consequences if doses are missed and toxicity if doses are in excess. In many cases, they have extreme polypharmacy, medical fragility, and reliance on complicated medication schedules and administration routes all typically managed by undersupported family caregivers [3,4]. A national sample found that CMC in the United States have nearly 5 times higher odds of an adverse drug event leading to an emergency department (ED) visit than other children, and more than 1 of 50 CMC ED visits are due to adverse drug events [5]. Administration discrepancies, which have been defined as inconsistencies in medication indication, dose, formulation, frequency, and route between caregivers and prescriptions, presumably lead to adverse drug events [6]. Such errors have been linked directly to preventable adverse drug events in children, including hospitalizations, ED visits, and morbidity [5-7].

Families who care for CMC experience at least 2 unmet needs that influence medication safety outcomes such as administration discrepancies [8]. First, there are no reliable and tested tools that support medication administration accuracy for families of CMC, despite the high risk and complex nature of CMC medication management [9]. Second, no tools exist to support families to ensure safe medication management across the network of all people involved in the 24-hour daily care that most CMC require. This network of secondary caregivers (SCGs) includes other family, in-home professionals, school aides, respite workers, and so forth. CMC networks are unstudied despite the impact caregiving network performance has on caregiver, patient, and health care system outcomes in other populations [10,11].

To address these gaps, we co-designed the mobile app, Medication Safety @HOME—Meds@HOME with CMC families, SCGs, and health professionals. Meds@HOME was designed specifically to meet challenges related to medication administration for CMC in the home and the community [12]: giving the right medication at the right time; communicating with others about medications; and accommodating complex medical routines. The app allows the primary caregiver (PCG), that is, the parent or the guardian primarily responsible for overseeing a child’s care, to enter a child’s medications and care needs into the app and then mark when activities have been completed. The app also allows users to (1) create a child profile (listing likes and dislikes, allergies, caregiver contact information, etc), (2) store care instructions (how to prepare medication, when to use a sick day plan, or what to do in certain health emergencies, etc), (3) receive reminders for upcoming and overdue care routines and medication refills, and (4) post alerts for other caregivers on the child’s care team. The PCG invites as many SCGs as they would like, for example, extended family or a school or home nurse, to also use the account. SCG can then also view the child’s profile and care instructions, receive reminders for upcoming and overdue care routines, and check off tasks as they are completed.

Although Meds@HOME was created using user-centered design methods [13], its impact on medication administration accuracy is unknown. This trial will evaluate the effectiveness of the Meds@HOME platform to improve medication accuracy for PCGs and SCGs. The research question is, “For children with medical complexity who receive at least one high-risk medication, does use of the Meds@HOME digital intervention improve medication administration accuracy for primary caregivers as compared to usual care over 6 months?” We hypothesize that Meds@HOME will improve caregivers’ medication administration accuracy by creating standardized medication management across the group of individuals caring for a child.

### Objectives

This study aims to evaluate the effectiveness of Meds@HOME on medication administration accuracy for PCGs and for SCGs.

## Methods

### Study Design

This randomized controlled trial will test the hypothesis that medication administration accuracy is improved for caregivers who use the Meds@HOME mobile app within the caregiving networks of CMC aged between 0 and 16 years who use high-risk medications. Participant accrual will occur over 12 months at 1 site, with participant’s enrollment duration lasting 6 months.

### Participants and Setting

The study will be conducted at 1 site: the University of Wisconsin (UW) Health Kids American Family Children’s Hospital affiliated with the UW School of Medicine and Public Health, United States. We will recruit 152 PCGs, 152 children, and up to 304 SCGs over 12 months. If there is more than 1 PCG for a child, the other PCG may be invited to participate in the study as a PCG as well. If this occurs, the total number of PCGs will exceed 152 participants. The duration of the study for each participant will be 6 months.

The study population will consist of (1) CMC prescribed at least 1 scheduled high-risk medication and receive care at UW Health, and (2) their caregivers. The study distinguishes between 2 types of caregivers: (1) PCGs (child’s parent or legal guardian), and (2) SCGs (others who regularly care for the child, defined in the section Inclusion Criteria). Potentially eligible children will be identified by an analyst, who is not a part of the research team, by querying the electronic health record (EHR) data warehouse using diagnostic codes and the eligibility criteria.

### Inclusion Criteria

Participants are caregivers of CMC who take 1 or more high risk medications. Children, PCGs, and SCGs must meet all inclusion criteria to be eligible to participate in the study.

Child eligibility criteria include (1) providing assent, if appropriate; (2) being aged between 0 and 16 years at the start of study; (3) having 2 or more different complex chronic conditions [14]; (4) having had at least 2 or more encounters in the American Family Children’s Hospital system; and (5) having at least 1 active outpatient prescription for a scheduled high-risk medication. High-risk medications are defined from prior literature [6,9,15-17] and include the following: antiepileptics, opioids, tone or spasticity medications, psychotropics, stimulants, anticoagulants, sleep aids, antiarrhythmics, pulmonary hypertension medications, and immunosuppressants. Medications must be *active* (currently being taken) and chronic, defined as a 90-day script or a 30-day fill plus 2 refills. As needed or PRN medications are not included.

PCG eligibility criteria include (1) written informed consent, (2) willingness to comply with study procedures and availability for the duration of the study, (3) age of 18 years or older, (4) comfort speaking and reading in English, (5) self-identifies as a PCG of the study eligible CMC, (6) providing care on an ongoing basis to the study eligible CMC in the home, and (7) having an iOS or android mobile device (smartphone or tablet) with a phone plan that includes daily Wi-Fi service and data.

SCG eligibility criteria include (1) identification by the PCG as a “secondary caregiver,” (2) written informed consent, (3) willingness to comply with study procedures and available for the duration of study, (4) 18 years of age or more, (5) comfort speaking and reading in English, (6) providing care on an ongoing basis to the study eligible CMC, (7) administering medications to the study eligible CMC, and (8) having an iOS or android mobile device (smartphone or tablet) with a phone plan that includes daily Wi-Fi service and data.

Exclusion criteria for PCGs are having another child from the household who is already enrolled in the study in order to avoid the clustering effects of potentially having both multiple PCGs and multiple children within the same household. Otherwise, to maintain broad inclusivity in the study, the exclusion criteria for PCGs and SCGs are limited only to failing to meet each inclusion criterion.

### High-Risk Medication Determination

Data on medication use are abstracted from the outpatient prescribing records in the EHR data warehouse by an analyst who is not a part of the research team. Data abstracted for all high-risk medications for all potentially eligible children include study ID, medication name (generic and brand), indication, dose, formulation, frequency, route, and high-risk medication category indicators (ie, antiepileptic, opioid, tone or spasticity, etc), and a randomly generated number. The medication database is updated monthly, so that study staff have access to current prescription orders throughout the recruitment period.

To avoid introducing sampling bias into the process of selecting a patient-specific high-risk medication for outcome assessment, a systematic, hierarchical procedure was designed for implementation prior to randomization. First, the research team rank-ordered high-risk medication categories from those perceived to be most to least prevalent and at highest risk for adverse events if incorrectly administered. The order is as follows (Textbox 1).

For children who receive multiple high-risk medications, study outcome assessments focus on 1 randomly selected medication from the highest category. Within each category, high-risk medications are arranged in random sequence using the random number assigned by the analyst. For example, if a child takes 3 antiepileptics, the study staff identifies the first high-risk medication in random order and confirms with the PCG that the child still takes this medication. If the child no longer takes the medication, the staff moves sequentially down the randomly ordered list until a medication the child is taking is identified. If no high-risk medications are confirmed by the PCG, then the child is deemed ineligible.

**Medication determination order from highest to lowest risk.**

High-risk medication determination order:

1. Antiepileptics
2. Opioids
3. Tone or spasticity medications
4. Psychotropics
5. Stimulants
6. Anticoagulants
7. Sleep aids
8. Antiarrhythmics
9. Pulmonary hypertension medications
10. Immunosuppressants

### Enrollment

Potentially eligible PCGs will be mailed an opt-out letter describing the study and then contacted 1 week later by telephone to screen for eligibility and interest. Eligible and interested PCGs will be scheduled for a formal enrollment visit. Enrollment visits will be conducted via a web-based teleconference in a quiet, private area offering confidentiality. The enrollment visit will be considered T_0_, and study enrollment will continue for 6 months from that date. First, PCG consent (and child assent) will be obtained. Staff will also conduct the following tasks.

#### Baseline Primary Outcome Assessment

Study staff will use a primary outcome assessment case report form and scripted interview to elicit and record PCGs’ account of their child’s high-risk medication prescription information. After the PCG is presented the name (generic or trade) of the high-risk medication as described in Textbox 1, PCGs will be asked via a standard script to describe the medication’s indication, formulation, dose, frequency, and route. All answers are recorded verbatim, and families are asked not to refer to medication bottles or written materials.

#### Identification of SCGs

The PCG will identify SCGs and rank them from most to least involved with medication delivery. SCGs will be approached in that order until up to 2 have been enrolled per child. They will first be invited to the study via an opt-out letter that can come from either the PCG or the study staff, based on PCG preference. Once they receive an invitation, SCGs can indicate interest in participation through one of the following ways: (1) completing an web-based interest form using a hyperlink provided in the opt-out letter, (2) waiting for study staff to follow up by phone to discuss the study 1 week after the opt-out letter is sent, or (3) contacting study staff by phone or email to discuss the study.

#### Randomization Into a Study Group

Participants will be randomized into either the control group or the intervention group. The study will use a 1:1 allocation with random block sizes of 2 and 4 [18]. Block randomization will be achieved with a computer-generated random number list prepared by the study biostatistician with no clinical involvement in the trial. Only the biostatistician will have access to the table listing the randomly allocated block sizes and sequence of group assignments; study staff will not. This ensures balanced allocation to the intervention and control groups while maintaining allocation concealment for study staff [19]. Randomization will be stratified by enrollment status in the UW Pediatric Complex Care Program (ie, enrolled or not) because the additional health care support and education this population receives could influence outcomes and intervention use. SCGs will be assigned to the same group as the PCGs.

If assigned to the intervention group, staff will help the PCG download the app, create an account, invite a caregiver, and set up an initial routine.

After the enrollment visit, both PCGs and identified SCGs will be asked to complete a self-administered questionnaire (SAQ) using REDCap (Research Electronic Data Capture; version 14.2.2). The questionnaire will be emailed to both PCGs and SCGs following the PCG enrollment and expressed SCG interest (through either completion of the SCG interest survey or phone discussion with study staff). For PCGs, the questionnaire includes questions about general caregiving as well as medication delivery knowledge, attitudes, and practices, and the PCGs’ confidence in their caregiving network, that is, all SGCs. For SCGs, the questionnaire includes questions about demographics, comfort with medical information, and for the identified high-risk medication, its indication, formulation, dose, frequency, and route. A similar questionnaire will be emailed at the study exit.

### Description of the Intervention (App Use)

Participants randomized to the intervention group will be assigned to use the Meds@HOME app for 6 months. Meds@HOME is a software application designed for use on a personal mobile device (such as phone or tablet). It is available on iOS and Android operating systems (Multimedia Appendix 1).

The app has the following core functionalities, which are managed by the child’s PCG.

#### Caregiver Profile

To establish an account, specific profile fields are required, including name, email, phone number, relationship to child, caregiver photograph, and preferred method of communication. PCGs can invite as many other caregivers (eg, extended family, home nurse, and school aide) to use the app as they wish. They can also deactivate any caregivers. Based on co-design priorities set by caregiver stakeholders, the system was not designed for use by the child’s hospital, primary, or specialty clinical team members.

#### Child Profile

At a minimum, the child’s profile must include their name. Other optional fields include photograph, gender, date of birth, address, and “important things to know about me” (allergies, I’m calmed by, I’m upset by, I need assistance with, best way to communicate with me, comfort measures I prefer, and my technology). 

#### Caregiving Routines and Routine Tracking

The app allows PCGs to create custom daily routines, for example, “Afternoon Meds,” “Lunch,” or “Prep for next day,” and so forth*.* Routines can include general caregiving tasks, medication details, meal instructions, and so forth. PCGs can detail how to perform the routine, start dates and times, and recurrence frequency, such as daily, weekly, and monthly. Customizable push notifications can alert caregivers to upcoming routines in the manner they wish. Only PCGs can create, edit, and delete routines. All caregivers can mark routines as complete and receive notifications.

#### Inventory Reminders

For medications, caregiving supplies, and foods, PCGs can set notification intervals for refill reminders.

Most functionalities are optional or voluntary and can be left blank. A child’s PCG determines the type and level of detail to input and who is invited to use the app.

### Description of the Control Group (No App Use)

PCGs randomized to the control group will be asked to complete the SAQ at the beginning and end of the 6-month trial, similar to the intervention group. SCGs in the control group will not use the Meds@HOME app. During the 6-month time frame, PCGs and SCGs will continue their normal caregiving routines.

### Conclusion of the Study

**Figure 1:**
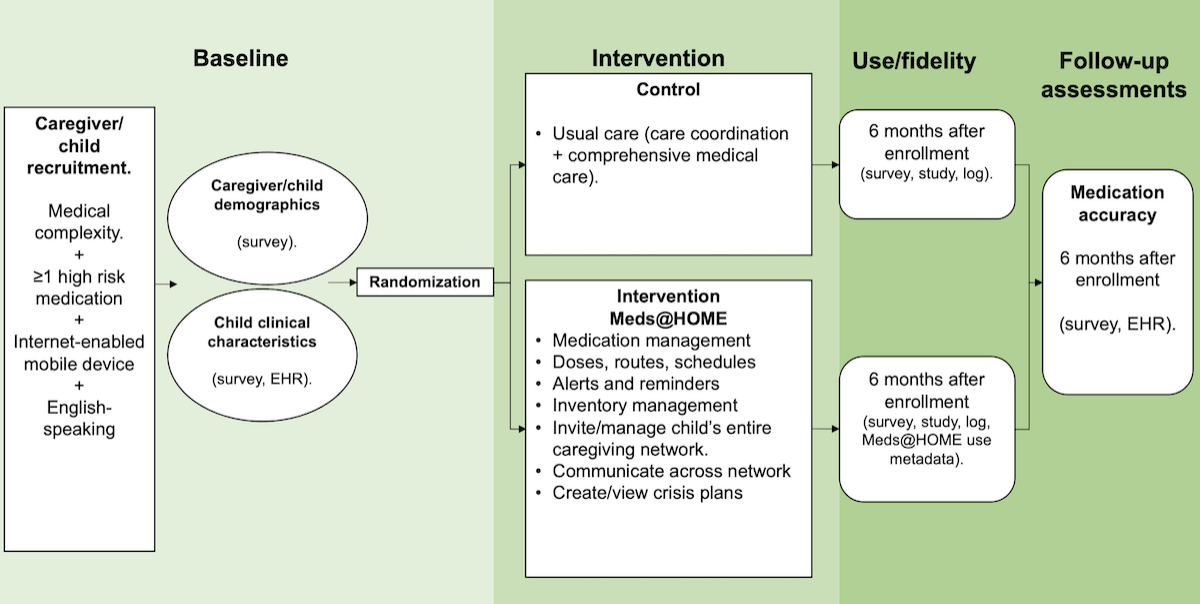
Meds@HOME study randomized controlled trial design. EHR: electronic health record.

At the conclusion of the study, PCGs and SCG will be emailed via REDCap a follow-up SAQ that is identical to the one received at the beginning of the trial. Study staff will follow up with PCGs via phone to conduct an exit primary outcome assessment. The high-risk medication random selection and assessment procedures are the same at study entry and exit. The same medication can, but does not have to, be used for both outcome assessments. A PCG is considered to have completed the study if baseline and exit POA and SAQs are completed, while a SCG must complete the baseline and exit SAQs (Figure 1 and Table 1).

**Table 1:** Schedule of activities of the Meds@HOME Trial-Plan for assessment of intervention at the beginning and end of the study period, with the depiction of staff involved.

				Study period, *T*=X months (staff involved)		
			Prior to enrollment visit (research coordinator)	Enrollment visit, *T*=0 months (research coordinator)	Exit visit, *T*=6 months (research coordinator)	Assessment, *T*>6 months (blinded assessor)
**PCG^a^ activities**						
	Phone screen and eligibility		✓			
	Completion of SCG^b^ worksheet		✓			
	Confirm eligibility			✓		
	Informed consent			✓		
	Child assent (if applicable)			✓		
	SCG individual identification			✓		
	**Randomization**			✓		
		Baseline SAQ^c^		✓		
		Primary outcome assessment		✓	✓	✓
		Exit SAQ			✓	
		Participant payment		✓	✓	
**SCG activities**						
		Baseline and outcome SAQ		✓		
		Exit and outcome SAQ			✓	
		Primary outcome assessment				✓
		Participant payment		✓	✓	

^a^PCG: primary caregivers.

^b^SCG: secondary caregivers.

^c^SAQ: self-administered questionnaire.

### Ethical Considerations

This study received approval from the UW institutional review board on January 19, 2022 (2021-1532). All participants will provide informed consent before taking part in the study. Informed consent materials will be provided in private spaces in both written and verbal formats and the study design will be reviewed in detail. Review of study design will include random assignment to the intervention and control groups, potential risks of participation, protections against risk, and the rights of human research participants. Parents or children can revoke their consent or assent at any point. Any identifying information kept for the purpose of contacting participants or linking data over time will be kept secure, in REDCap, a locked filing cabinet, or in a password-protected electronic file, and will be destroyed when the study is complete. Data will be de-identified at study conclusion. The study’s data and safety are monitored every 6-12 months by the data-monitoring committee at the UW Institute for Clinical and Translational Research. All PCGs will receive an incentive of US $200, divided in 2 parts: US $100 at enrollment and US $100 after the exit survey, in the form of a gift card, check, or cash. SCGs will receive an incentive of US $150, divided into 2 parts: US $75 at both enrollment and after exit survey completion.

### Outcomes

#### Primary End Point

The primary outcome is medication administration accuracy, defined dichotomously as correct identification of all the following for a selected patient-specific high-risk medication: indication, formulation, dose, frequency, and route of administration (Table 2).

**Table 2:** Primary and secondary objectives of the study with corresponding end points being used for measurement.

Objectives			End points		Number of items and estimated time to complete
**Primary**					
	To evaluate the effectiveness of Meds@HOME on PCG^a^ medication administration accuracy [6].	Rate of medication administration accuracy measured dichotomously as correct identification of each of the following for a randomly selected high-risk medication: indication, formulation, dose, frequency, and route at baseline and after 6 months.		5 items, approximately 5 minutes.	
**Secondary**					
	To evaluate the effectiveness of Meds@HOME on SCG^b^ medication administration accuracy [6].	Rate of medication administration accuracy, measured as in the primary outcome, among SCGs at baseline and after 6 months.		5 items, approximately 5 minutes.	
	To evaluate Meds@HOME’s effect on ADE^c^ hospital use [5].	Count of UW^d^ hospital encounters during study period with ADE codes.		1 item, extracted from electronic health record diagnostic and procedure codes.	
	To evaluate Meds@HOME’s effect on ADE UW ED^e^ visits [5].	Count of UW ED encounters during study period with ADE codes.		1 item, extracted from electronic health record diagnostic and procedure codes.	
	To evaluate Meds@HOME’s effect on parent-reported medication adherence [20,21].	Mean PCG-reported medication adherence using the Adherence to Refills and Medications Scale (ARMS) after 6 months.		12 items, approximately 5 minutes.	
	To evaluate Meds@HOME’s effect on medication activation [22].	Mean Family Caregiver Activation in Transition (FCAT) 5 medication-specific items—composite and individual items after 6 months.		5 items, approximately 2 minutes.	
	To evaluate Meds@HOME’s effect on parent-reported medication confidence [6].	Mean composite score after 6 months.		6 items, approximately 3 minutes.	
	To evaluate Meds@HOME’s effect on parent-reported medication understanding [6].	Mean composite score after 6-months.		5 items, approximately 2 minutes.	
	To evaluate Meds@HOME’s effect on all-cause hospital use.	Count of hospital encounters and hospital days during study period.		1 item, extracted from electronic health record encounters.	
	To evaluate Meds@HOME’s effect on all-cause ED use.	Count of ED encounters during study period.		1 item, extracted from electronic health record encounters.	
	To evaluate Meds@HOME’s effect on mortality.	Count of deaths during the study period.		1 item, 1 item, extracted from electronic health record encounters or study records.	
	To evaluate Meds@HOME’s effect on the primary outcome measured as 5 individual components.	Rate of individual components each measured dichotomously (indication, formulation, dose, frequency, and route); mean of individual components after 6 months.		No new items, this disaggregates the primary end point.	

^a^PCG: primary caregivers.

^b^SCG: secondary caregivers.

^c^ADE: Adverse drug event.

^d^UW: University of Wisconsin.

^e^ED: emergency department.

### Assessment Procedures

The medication administration accuracy measure and many of the caregiving measures have been documented as reliable in prior literature; however, few validation studies have been conducted with these measures [22-27]. We will ensure reliability in data collection through direct observation, data auditing, establishing clear data dictionaries and definitions, using uniform variable definitions, and a central data repository coordinated and maintained at UW. The primary and secondary end points are measured following completion of the PCG enrollment and exit primary outcome assessment case report forms and SCG SAQs. The most recently abstracted EHR prescription data for the selected high-risk medication is considered the gold standard. Following data collection, a clinician with expertise in care for CMC and high-risk medication management who is blinded to treatment assignment (*blinded outcome assessor*) compares caregiver responses with the gold standard data. The blinded outcome assessor scores participant responses for each component of medication administration accuracy (ie, indication, dose, formulation, frequency, and route) as correct, incorrect, or missing. Each component must be correct to meet the study end point.

To establish the reliability of the primary end point assessments, the first 25 cases are independently, dually coded by 2 blinded outcome assessors. The study biostatistician calculates interrater reliability using kappa for the primary end point (ie, the dichotomous composite measure of medication administration accuracy). If k value is ≥0.85, the coders have strong, almost perfect agreement [28,29] and the remaining data are single coded. If k value is <0.85, discrepant items are recoded, the outcome assessors are retrained, and the next 25 cases are again independently, dual-coded. Kappa is recalculated for the next 25, and the same procedures are completed until either k value is ≥0.85 or data collection is complete.

### Data Collection, Storage, and Protection

All data will be collected via phone scripts, EHR abstraction, standardized SAQs, and case report forms completed by trained study staff. All data for this study will be housed in REDCap, managed by UW’s Institute for Clinical and Translational Research as a 21 Code of Federal Regulation Part 11-compliant data capture system [30]. REDCap includes password protection and internal quality checks, such as automatic range checks, to identify inconsistent, incomplete, or inaccurate data. Clinical data will be entered directly from the source documents or entered directly through secure SAQs emailed via REDCap surveys to participants.

Data from the Meds@HOME app will be stored in databases on the UW School of Medicine and Public Health Department of Pediatrics secure servers. The UW Department of Pediatric servers follow all UW campus and UW Health privacy and compliance requirements. Access to study folders will be limited to study staff with appropriate training and permissions.

### Sample Size Considerations

We will enroll 152 PCG participants. The study is powered to detect an anticipated clinically important difference in the primary end point (medication administration accuracy rate). Based on preliminary data on medication administration accuracy, the observed rate was 41% in PCGs [31]. Hence, the anticipated medication administration accuracy rate for the control arm of this study is 41%. We estimate that Meds@HOME use will increase the outcome rate from 41% (control arm) to at least 70% (intervention arm). This increase is considered a clinically important improvement. The sample size (n=152) will detect this difference in the medication administration accuracy rates between arms, calculated for 90% power at the 2-sided .05 significance level based on a *z* test with continuity correction [32]. Recruitment needs are feasible based on a projected eligible population (n=1100), an estimated 20% enrollment rate, and a 10% loss to follow-up rate. Furthermore, we estimate that 2 caregivers may provide independent responses for up to 5%-10% of the households. With a proposed sample size of 152 households, the expected number of PCGs is between 160 and 167. With this sample size, the anticipated difference of 41% versus 70% in the medication accuracy rates will be detected with at least 94% power at the 2-sided .05 significance level, based on generalized estimating equation analyses, assuming an intraclass correlation coefficient (for up to 2 PCGs within the same household) of 0.05-0.50 [33].

With respect to secondary outcomes, the proposed sample size will also provide 82%-99% power to detect moderate (Cohen *d*=0.5) to large (Cohen *d*=0.8) effect sizes at the 2-sided .05 significance level in secondary outcomes between arms [28,29]. Because we assume that PCG will perform better than SCG, the anticipated medication administration accuracy rate is assumed to be <41% in the SCG cohort. If the SCG sample’s control group performance was only 30%, we would have 90% power to detect an increase to 60% with only 136 enrollees. Therefore, with our planned enrollment of 152 (ie, 1 SCG for every PCG), we will have adequate power to conduct the SCG analysis even with poorer enrollment or smaller effect sizes than expected (Table 3).

**Table 3:** Sample size requirements for detecting differences in the medication administration accuracy rates between arms with 90% power at the 2-sided .05 significance level.

Sample sizes	Intervention group outcome, n				
	Medication administration accuracy = 60%	Medication administration accuracy = 65%	Medication administration accuracy = 70%	Medication administration accuracy = 75%	Medication administration accuracy = 80%
Final total sample needed	300	194	136	96	74
Final sample per treatment arm	150	97	68	48	37
Eligible subjects to achieve sample	370	240	168	120	91
Enrolled subjects to achieve sample	334	216	**152**	108	82

### Statistical Analysis Plan

Given that the intervention being tested requires caregiver use to achieve success, we will use an intention-to-treat analysis approach [18]. We plan to use the primary outcome data to assess Meds@HOME’s impact on medication administration accuracy of PCGs of CMC measured at study baseline and exit 6 months later. This binary measure is derived from prior studies involving CMC PCGs [6] and includes demonstrating parent recall of complete medication instructions for 1 patient-specific high-risk medication, compared with prescription details. Medication administration accuracy is defined dichotomously as correctly identifying all the following for a selected patient-specific high-risk medication: indication, formulation, dose, frequency, and route of administration. The outcome will be evaluated as the change in percentage of intervention participants compared with control participants demonstrating medication administration accuracy at 6 months compared with baseline. Primary analysis will test differences between treatment (intervention or control) groups in the primary and secondary outcomes. For the primary analysis, univariate generalized estimation equation analysis [33] with exchangeable correlation structure to account for potentially 2 PCGs responding from the same household will be conducted to compare the medication administration accuracy rates between study arms. The effect size of the difference in medication administration accuracy rates will be quantified by calculating the odds ratio, which will be reported along with the corresponding 95% CI. Furthermore, multivariate generalized estimation equation analysis [33] will be performed to compare the medication administration accuracy rates between study arms. In this analysis, clinical and demographic characteristics will be included as covariates in an initial nonparsimonious model. Collinearity will be evaluated and the least absolute shrinkage and selection operator and elastic net penalty methods for logistic regression models will be used to identify a parsimonious model with independent covariates.

The secondary outcome count variables include the numbers of adverse drug event ED visits and hospitalizations and total numbers of all-cause hospital days, hospitalizations, and ED visits. This will be analyzed using univariate and multivariate mixed-effects negative binomial regression models with household-specific random effects to account for overdispersion in the count data and responders from the same household. The cumulative number of these outcomes over the 6-month follow-up period will also be analyzed using a univariate linear mixed-effects model with household-specific random effects. In a secondary analysis, a multivariate linear mixed-effects model will be used where clinical and demographic baseline characteristics will be included as covariates and the least absolute shrinkage and selection operator method will be used to identify a parsimonious model. Secondary outcome binary variables, that is, individual components of the primary outcome, items from the medication activation, understanding and confidence measures answering, *strongly agree*, parent-reported medication adherence, and death rates, will be modeled in the same manner as the primary analysis. Each of these binary outcomes will be documented at final assessment and changes within and between study arms will be analyzed using univariate and multivariate generalized linear mixed-effects modeling with household-specific random effects.

Baseline comparisons of demographic variables and clinical characteristics will be conducted using a chi-square test (categorical variables) or a 2-sample test and nonparametric Wilcoxon rank sum test (continuous or quantitative variables) [34,35].

Because some participants may not use the Meds@HOME intervention, we will conduct a secondary analysis repeating all primary and secondary end point assessments using a per-protocol population analysis approach to complement the intention-to-treat approach [18]. The per-protocol population will be eligible participants who were randomized and achieved a level of compliance, defined as creation of at least 1 routine and 6+ log ins, 3 of which occurred in last 3 months of the enrollment period. The login number was chosen to reflect at least 1 login per month, with use throughout the intervention period.

To evaluate the impact of missing values (eg, due to loss of follow-up, incomplete data collection) we will conduct a sensitivity analysis by comparing the results obtained from the complete case analysis with the results obtained by imputation-based analyses. Specifically, multiple imputation will be used to impute the missing values of the primary and secondary clinical outcomes. For monotonic missing values data structures, we will use regression-based multiple imputation techniques. By contrast, we will use Markov Chain Monte Carlo–based imputation techniques for nonmonotonic missing value data structure.

## Results

**Figure 2:**
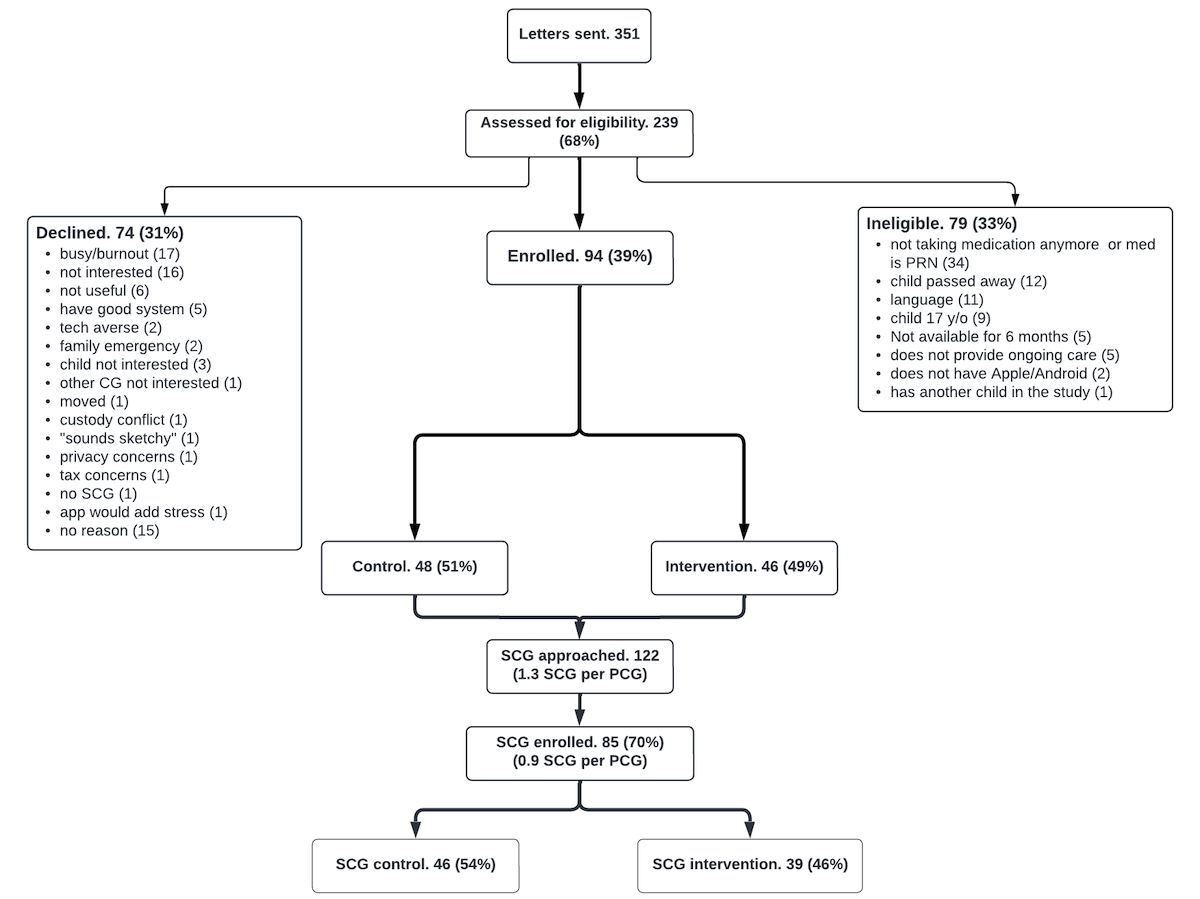
CONSORT (Consolidated Standards of Reporting Trials) diagram for Meds@HOME. CG: caregiver; PCG: primary caregiver; PRN: pro re nata (as needed); SCG: secondary caregivers.

This study was funded in May 2022. Initial institutional review board approval for this study occurred on March 9, 2023. Recruitment began on November 29, 2023. Data collection began on December 11, 2023. As of May 15, 2024, we have enrolled 62% (94/152) of PCGs (Figure 2). We expect recruitment to end by September 1, 2024, and the final participant will complete the study by January 28, 2025, at which point we will start analyzing the complete responses. We expect publication of results in the winter of 2025.

## Discussion

### Summary

The Meds@HOME mobile app is hypothesized to improve caregivers’ medication administration, reduce preventable adverse drug events, and ultimately improve chronic care management [36]. Previous research indicates that caregivers for CMC experience multiple challenges related to medication administration, including giving the right medication at the right time, communicating with others about medications, and accommodating complex and sophisticated caregiving routines [12]. The Meds@HOME mobile app offers a solution to each of these challenges. As a result, we anticipate that compared with baseline medication administration accuracy, both PCGs and SCGs using Meds@HOME will have statistically significant increases in this measure compared with the control group.

Compared with other interventions, Meds@HOME was developed from the expert perspectives of PCGs and SCGs for CMC, as well as clinicians [12]. There is currently substantial interest in using mobile health technologies to improve CMC care [37-39]. Meds@HOME is also unique compared with other interventions because of its focus on medication safety in the home and on coordinating care among multiple caregivers in addition to parents. Meds@HOME has the potential to decrease medication errors for CMC and promote confidence and connection within a caregiving network. Such outcomes will be evaluated through the study’s planned secondary outcome analyses. Importantly, Meds@HOME is a potentially scalable intervention that could be rapidly disseminated beyond the single site if efficacious. In this or future studies, Meds@HOME may also demonstrate broader improvements in CMC health, such as ED and hospital use. Following this randomized controlled trial, we intend to conduct real-world effectiveness and implementation research across multiple sites, with the goal of creating a tool that is widely available and promotes the health and safety of CMC.

### Limitations

Although we anticipate having adequate power to assess for intervention efficacy, this study still has limitations. First, while assessors are trained and blinded, the evaluation of medication administration accuracy has some subjectivity that could influence reliability and validity. This risk will be minimized by the dual-coding procedures described in the section “Assessment Procedures.” Although this study design introduces the possibility of interview bias, this risk is minimized by providing standard scripts embedded into for study staff to use when interacting with participants. Participants cannot be blinded to the intervention, and caregivers in the control and intervention groups will be aware of which they have been assigned to. We will attempt to minimize the risk of participant reactivity by not sharing the randomly selected high-risk medication ahead of time and by requesting participants not to refer to written materials or pill bottles during assessments. Finally, the limited ability to develop Meds@HOME in multiple languages requires participants to be comfortable reading and speaking English, limiting external validity. Future research will include software iterations in multiple languages to reach families with more geographic and culturally diverse backgrounds.

### Conclusions

Despite the limitations, this detailed study protocol provides a real-world, promising strategy for improving PCG medication safety for children who are taking high-risk medications and novel procedures for recruiting SCGs of CMC. This intervention may have extended positive impact by improving SCG medication accuracy, improving caregiver medication understanding and confidence, and influencing hospital and ED use as well as other health outcomes. In the future, this app could be used more extensively by caregivers and youth who are navigating complex medication routines, promoting medication safety among diverse caregiving networks.

## Appendix

Multimedia Appendix 1 Example of application screens.

## Abbreviations

CMC: Children with medical complexity
ED: emergency department
EHR: electronic health record
PCG: primary caregiver
REDCap: Research Electronic Data Capture
SAQ: Self-administered questionnaire
SCG: secondary caregiver
UW: University of Wisconsin

## Data Availability

The data sets generated and analyzed during this study are available from the corresponding author on reasonable request and completion of a data use agreement and institutional review board–approved protocol. Due to risk to identifiability intrinsic to the study’s data collection plans, the data set will not be deposited in a publicly available repository.
