# Supplementary figures and images for: Effectiveness of a Mobile App (Meds@HOME) to Improve Medication Safety for Children With Medical Complexity: Protocol for a Randomized Controlled Trial
Source: JMIR Res Protoc. 2024 Sep 9;13:e60621. doi: 10.2196/60621 (PMC11420605; doi:10.2196/60621)

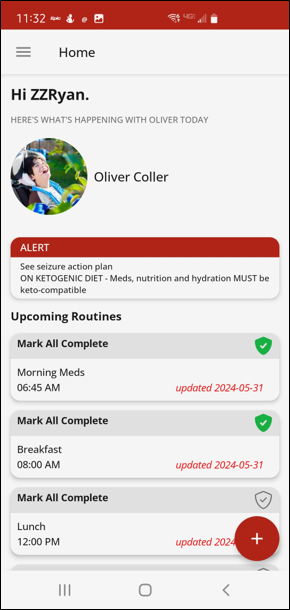
  
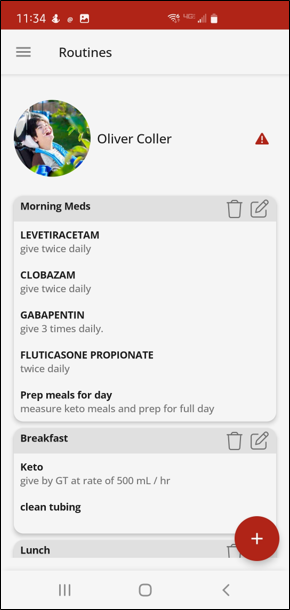
  
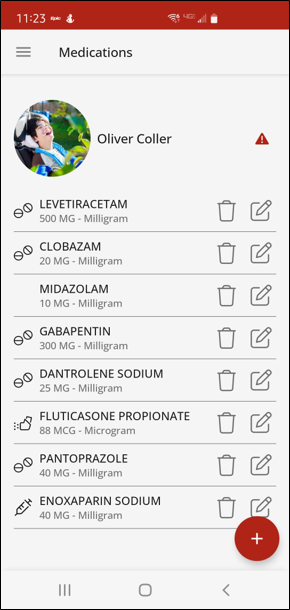
  
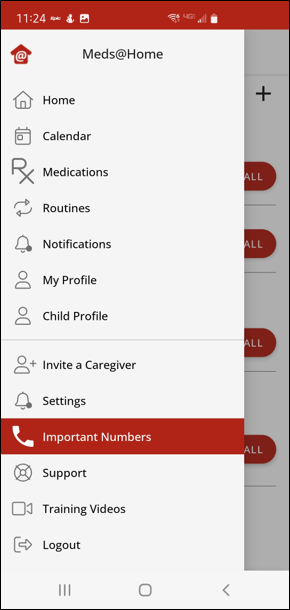
  
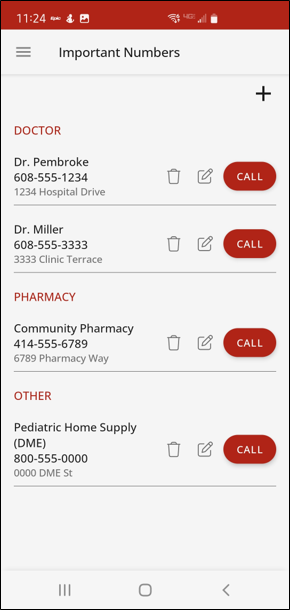
  
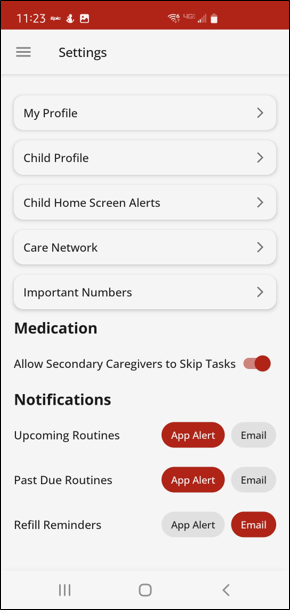

Supplement: Multimedia Appendix 1 [file resprot_v13i1e60621_app1.docx]
